# Exploring the potential of acceptance and commitment therapy model in self‐care behaviour in persons with heart failure

**DOI:** 10.1002/nop2.538

**Published:** 2020-06-11

**Authors:** Mohammed Munther Al‐Hammouri, Jehad A. Rababah, Mohammed Aldalaykeh

**Affiliations:** ^1^ Jordan University of Science and Technology Irbid Jordan

**Keywords:** acceptance and commitment therapy, committed action, heart failure, mindfulness, nursing

## Abstract

**Aim:**

This study examined the interaction among cognitive fusion, mindfulness and committed action on the self‐care behaviour in person with heart failure as guided by the acceptance and commitment therapy model.

**Design:**

An exploratory cross‐sectional design was used.

**Method:**

Participants with heart failure from in‐patients setting (*N* = 165) were recruited from two major regional hospitals. Data were collected using self‐report questionnaires of the study variables and demographic characteristics. Data were analysed using Hayes SPSS process macros.

**Results:**

Based on the acceptance and commitment therapy model propositions, all main and interaction effects, except for cognitive fusion, were significant. The current study showed that the main effect of the study variables on self‐care behaviour was insufficient and adding complex interactions between these variables improved the model fit, as it was suggested by the acceptance and commitment therapy model.

## 1 INTRODUCTION

Heart failure is a chronic condition that affects older people and affects the heart's ability to pump out blood through remodelling of the heart muscle (Huynh, Whitmore, Negishi, Marwick, & ETHELRED Investigators, 2019). Remodelling is followed by a compensation mechanism that further changes and damages the heart cells (Twedell, 2007). Thus, disease progression and exacerbations can go from bad to worse if preventive measures are not adopted. Heart failure is one of the most debilitating diseases affecting older people (Amouzeshi, Safajou, Kazemi, & Kianfar, 2019; Grady, 2008; Salyer, Schubert, & Chiaranai, 2012) and is associated with high mortality, morbidity and healthcare expenditure. For example, the number of persons with heart failure in the United States of America is about 6.5 million and half of them die within five years of diagnosis (Centers for Disease Control & Prevention, 2019). The estimated healthcare costs and lost productivity for persons with heart failure were $30.7 billion in 2012 (Centers for Disease Control & Prevention, 2019). It has been reported that heart failure is affecting about 40 million persons globally in 2015 (Vos et al., 2015). Considering the serious impact of heart failure on personal and societal levels, we should aim at improving disease outcomes and slow down the progression of the disease. One way to do that is through self‐care behaviour to control symptoms and disease progression (Macabasco‐O'Connell, Crawford, Stotts, Stewart, & Froelicher, 2008). Yet self‐care behaviour in persons with heart failure is still below optimum due to lack of adherence to the treatment regimen (Bidwell et al., 2015; Herber, Atkins, Störk, & Wilm, 2018; Riegel, Driscoll, et al., 2009).

### 1.1 Background

Self‐care in persons with heart failure can be defined “as a process of maintaining health through health‐promoting practices and managing illness” (Jaarsma, Cameron, Riegel, & Stromberg, 2017, p. 71). Self‐care has been reported to minimize hospitalizations and deaths related to heart failure (Artinian, Magnan, Sloan, & Lange, 2002; Centers for Disease Control & Prevention, 2019; Lee, Moser, Lennie, & Riegel, 2011; Macabasco‐O'Connell et al., 2008). In addition, self‐care behaviour improves health, prevents diseases and restores health (Arcury et al., 2009; Macabasco‐O'Connell et al., 2008). Thus, several studies have examined the factors associated with self‐care in persons with heart failure. For example, self‐care was associated with higher education, lower symptom severity, greater comorbidity, less depression and lower self‐care confidence (Cameron, Worrall‐Carter, Riegel, Lo, & Stewart, 2009; Holzapfel et al., 2009; Schnell‐Hoehn, Naimark, & Tate, 2009). Some studies investigated statistical models to predict self‐care in persons with heart failure (Cameron et al.; Rockwell & Riegel, 2001). However, these studies are still unable to provide an in‐depth understanding to predict or promote self‐care to the optimum level, due to lack of adherence to the treatment regimen (Herber et al., 2018). One of the explanations for the limited effectiveness of some interventions targeting self‐care is that they are “neither theory‐based nor well defined” (Herber et al., 2018, p. 1). Thus, this study adopted the acceptance and commitment therapy (ACT) model to guide the study of self‐care behaviour in persons with heart failure.

The ACT model is a newly emerging model to guide behavioural interventions that aims at positive behavioural change (Hayes, Strosahl, & Wilson, 2011). It is based on the Relational Frame Theory (RFT) first coined by Dr. Steve Hayes (See Hayes et al., 2011 for more details). The ACT model suggests that interaction among three main response styles is necessary for any behavioural change to occur. The ACT is a model that consists of three response styles: open, centred and engaged (Hayes et al., 2011). Under each response style, there are two main skills that are involved in any behavioural change process (Hayes et al., 2011).

The open response style consists of two main skills: acceptance and diffusion. Acceptance refers to the skills that we need to learn to counter the effect of experiential avoidance (Hayes et al., 2011). Experiential avoidance refers to the individual tendency to avoid effective behavioural solutions to avoid negative emotions related to it (Vilardaga, Hayes, & Schelin, 2007). Defusion refers to the skill needed to deal with the problem of cognitive fusion (Vilardaga et al., 2007), while cognitive fusion, in its simplest way, can refers to the way that verbal behaviour affects our response to life events (Vilardaga et al., 2007).

The centred response style consists of the present moment and self‐as‐context skills (Vilardaga et al., 2007). The present moment, as the name reveals, refers to the person's ability to live in the here and now by focusing on the current moment, which helps the person to live in the here and now by focusing on the current moment (Vilardaga et al., 2007). Self‐as‐context is a skill needed by any person to take efficient action or response to environmental events. Self‐as‐context refers to perspective‐taking, which reflects a person's ability to reconsider life events from different perspectives in terms of different persons, time and places (Hayes et al., 2011).

The third style, the engaged response style, consists of values and committed action. Value is the skill needed to overcome the effect of cognitive fusion and experiential avoidance (Hayes et al., 2011). Committed action refers collectively to a person's behaviours that both emerge from and result in value‐driven pattern behaviour. In other words, if you examine the person's behaviours, they will be guided by that person's set of values (Hayes et al., 2011). On the other hand, the behaviours of the same individual will be consistent with the set of values for that person (Hayes et al., 2011).

The power of any psychological intervention lies in its ability to produce sustained behavioural change (Zhang et al., 2018). In other words, applying any intervention must first promote a behavioural change and, second, help in maintaining that behavioural change over time. There have been recent calls for theory‐based interventions that are more likely to improve the health outcomes in persons with heart failure (Deek et al., 2016). Meanwhile, the literature shows evidence that the ACT model‐based interventions resulted in long‐term behavioural changes (Zhang et al., 2018). To study the potential of the ACT model, we examined three variables that represent three response styles depicted in the ACT model. These variables are cognitive fusion, mindfulness and committed action that represents open, centred and engaged response styles, respectively.

Cognitive fusion refers to the problem of how our language abilities affect how we respond to daily life events (Zhang et al., 2018). Those who suffer from cognitive fusion tend to be entangled in their thoughts and verbal rules generated from their earlier experiences (Ruiz, Suárez‐Falcón, Riano‐Hernández, & Gillanders, 2017). Cognitive fusion has been studied in relation to various human behaviours and health‐related variables. For example, cognitive fusion has been significantly and negatively associated with the quality of life in persons with diabetes (Samadifard & Mikaeili, 2016). Cognitive fusion was positively associated with negative health indicators, such as depression, anxiety, stress and post‐traumatic stress symptoms (Bardeen & Fergus, 2016).

Mindfulness is defined as “flexible, non‐defensive and present‐focused awareness” (McCracken & Yang, 2008, p. 480). Mindfulness has been associated with various human behaviour and health‐related issues. For example, mindfulness‐based interventions were associated with a reduction in anxiety and depression symptoms (Roemer, Williston, Eustis, & Orsillo, 2013), disordered eating habits and body image issues (Stumpf, 2017). On the contrary, mindfulness was associated with a better quality of life (Stumpf, 2017). Mindfulness was also associated with improved levels of well‐being, stress and psychological well‐being (Carmody & Baer, 2008).

Committed action can be defined as persistence on behaving in correspondence with personal values (Gagnon, Dionne, & Pychyl, 2016). Committed action has been associated with a reduction in self‐reported procrastination and depression (Gagnon et al., 2016). Committed action was also associated with better social functioning, mental health, vitality and general health controlling for the effect of pain and acceptance of pain (Gagnon et al., 2016).

## 2 THE STUDY

### 2.1 Aims

The purpose of the present study was to examine the interaction among cognitive fusion, mindfulness and committed action on the self‐care behaviour in a person with heart failure, as guided by the ACT model. This was achieved by examining the interaction among cognitive fusion, mindfulness and committed action that represents open, centred and engaged response styles in the ACT model, respectively, on the self‐care behaviour in the person with heart failure. The analysis was based on the relationships proposed by the ACT model. The future goal was to provide a conceptual basis for a more comprehensive application of the ACT model in persons with heart failure. The current study examines the following research question: Does the ACT model explain complex relationships affecting self‐care behaviour in persons with heart failure?

### 2.2 Design

This study is an exploratory cross‐sectional study.

### 2.3 Sample

Purposive sampling was used to recruit study participants. The inclusion criteria were a diagnosis of heart failure, at least 18 years of age and able to read and write to fill out self‐report questionnaires. The exclusion criteria were diagnosis of dementia. Data were collected from eligible participants with heart failure admitted to two major Jordanian hospitals. The data were collected using self‐report questionnaires.

The sample size was determined based on the expected effect size using multiple linear regression. Cohen reported that there are three main values of 0.02, 0.15 and 0.35, for small, medium and large effect sizes, respectively (Cohen, 1988). Thus, a sample of about 160 persons with heart failure was based on a small to medium effect size (0.05) and the power of 0.80 was deemed sufficient for the current study purpose. In the current study, we recruited 165 heart failure patients who completed and returned study questionnaires.

### 2.4 Data collection

This study was a cross‐sectional exploratory study. Data were collected from eligible participants using self‐report questionnaires during their hospital stay at a university hospital. After checking the inclusion criteria for each participant, the research assistant approached the potential participants and offered them the choice to participate in the study. The participants were given a choice to withdraw from the study at any time. For those who agreed to participate, a paper‐based study kit was handed to the participant. A research assistant was available all the time for any questions from the participants.

Self‐care behaviour was determined using a self‐care maintenance scale from the Self‐Care of Heart Failure Index Version 6.2 (SCHFI‐V6) (Riegel, Lee, Dickson, & Carlson, 2009). The self‐care maintenance scale in the SCHFI‐V6 consists of 10 items with a 4‐point Likert‐like response scale (Riegel, Lee, et al., 2009). Scores were standardized by converting it to a 100‐point scale. Higher scores reflect higher levels of self‐care behaviour. A cut‐off score of 70 out of 100 defines adequate self‐care behaviour. The psychometrics of the SCHFI‐V6.2 has been examined and found adequate. For example, in a sample of 154 persons with heart failure (Riegel, Lee, et al., 2009), the coefficient alpha was .55 for self‐maintenance. The low consistency parameter was justified by the low number of symptomatic patients in their sample (Riegel, Lee, et al., 2009). In addition, some researchers suggested not using the coefficient alpha to assess this self‐care maintenance scale reliability due to self‐care maintenance scale multidimensionality.

(Barbaranelli, Lee, Vellone, & Riegel, 2014). Its use in practice and research has been validated (Vellone et al., 2013).

Cognitive fusion is measured using a Cognitive Fusion Questionnaire (CFQ). Cognitive Fusion Questionnaire is 7 items with a 7‐point Likert‐like response scale from 7 = always; 1 = never true; the possible score range is 7–49. (Gillanders et al., 2014). A higher CFQ score indicates a higher level of cognitive fusion (Ruiz et al., 2017). The CFQ showed good psychometric properties. For example, Cronbach's alpha reported for CFQ for the English and Spanish versions ranged between 0.87–0.93 (Gillanders et al., 2014; Romero‐Moreno, Márquez‐González, Losada, Gillanders, & Fernández‐ Fernández, 2014; Solé et al., 2016).

Mindfulness was measured using the Mindful Attention Awareness Scale (MAAS) (Brown & Ryan, 2003). The MAAS is a 15‐item measure with a 6‐point Likert‐like response scale from 1(almost always)–6 (almost never) in relation to a respondent's everyday experience (McCracken & Yang, 2008). Mindful Attention Awareness Scale Cronbach's alpha above .80 (McCracken & Yang, 2008). To calculate a participant's score, we take the mean of the 15 items with higher scores, which reflect higher levels of dispositional mindfulness.

Committed action was measured using the Committed Action Questionnaire 8‐item version (CAQ‐8) (McCracken, Chilcot, & Norton, 2015). The CAQ is an 8‐item measure with a 7‐point Likert‐like response scale from 0 (never true)–6 (always true). After reversing negatively keyed items, a higher total score refers to higher commitment to action. The Cronbach's alpha, .87, of the CAQ‐8 supports the internal consistency of the measure (McCracken et al., 2015).

### 2.5 Ethical considerations

The IRB approval was obtained prior to the data collection. Informed consent has been obtained from the participants prior to their participation in the study. Patients were contacted and personally invited to participate in the study during their hospital stay. The participants had the option to withdraw from the study at any time.

### 2.6 Data analysis

**FIGURE 1:**
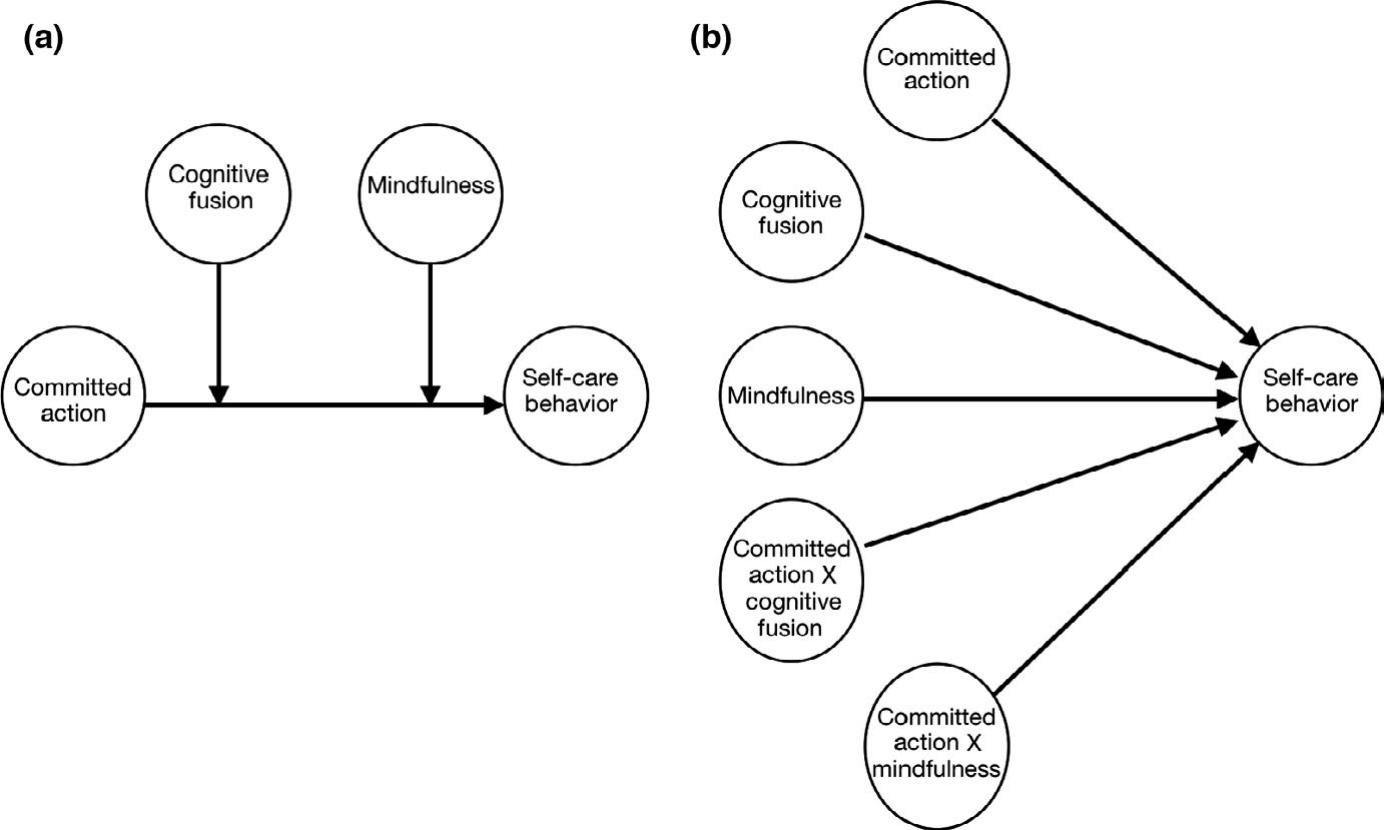
Conceptual and statistical models of the study adopted from model 2 Hayes (2013). (a) Theoretical model. (b) Statistical model

The data were analysed using SPSS® version 25 (IBM). First, we examined the descriptive statistics and bivariate relationships among study variables based on Baron and Kenny's guidelines for testing moderation and mediation relationships (Baron & Kenny, 1986). Then, we examined the main effects of mindfulness, cognitive fusion and committed action on self‐care maintenance. In addition, we ran Pearson's correlation, *t* test and one‐way ANOVA between the self‐care maintenance and participants’ age, gender and smoking and functional status and educational level. The correlation between self‐care maintenance and age was not significant. In addition, there were no significant differences in terms of self‐care maintenance based on gender, smoking, functional status and educational level. Thus, all of these variables were excluded from further analysis, as covariates, in the current study. Then, we re‐ran the same regression but added the interaction terms suggested by the statistical model that guided this study, which was derived from the ACT Model (See Figure 1). According to this model, we entered the interaction terms of committed action X mindfulness and committed action X cognitive fusion to the previously tested model of the main effects of the study variables. The linear regression was done to see whether the newer model with the interactions proposed by the ACT model would improve the regression model fitness. Finally, the decision was made to use Andrew Hayes macros if any of the interaction terms turned out to be significant (Hayes, 2013). This final step would examine the nature and the direction of the interaction among study variables. Andrew Hayes has provided plugins in the SPSS to examine the complex relationships among variables affecting human behaviours. According to Hayes (2013), human behaviour is a complex phenomenon that cannot be captured by simple and direct relationships. Rather, instead, Hayes recommends use of moderation, mediation and conditional processing analyses that could be more useful and insightful in explaining complex human behaviour. For more details about his proposed methods, the reader is advised to read Hayes (2013).

### 2.7 Validity and reliability

The variables in this study were measured using well‐established and validated measures. Cronbach's alpha values for the used measures in our current sample were .87, .57, .89 and .58 for MAAS, CAQ‐8, CFQ and self‐care maintenance, respectively.

## 3 RESULTS

The mean age of our sample was 58.8 years (*SD* = 11.38), ranging from 31–87. Most of our sample was males (*N = *134). Most were non‐smokers (*N = *101). Other demographic and clinical characteristics are shown in Table 1. The average self‐care maintenance score was 43 (*SD* = 15.3), ranging from 6.6–79.9. Only six participants scored above 70; the cut‐off that defines adequate self‐care behaviour.

**TABLE 1:** Demographic and clinical characteristics of study sample (*N* = 165)

Variable	*n*
Previous admission due to heart condition exacerbation	
0	23
1	55
2	48
3	20
4 and more	19
Education	
Did not complete high school	36
High school diploma	76
Vocational or some college	21
College	32
Functional status	
Class I	12
Class II	69
Class III	61
Class IV	23

Self‐care maintenance was positively correlated with mindfulness (*r* = .19, *p* < .05) and committed action (*r* = .18, *p* < .05) but was negatively correlated with cognitive fusion (*r* = −.21, *p* < .01). Cognitive fusion was negatively correlated with mindfulness (*r* = −.19, *p* < .05) and committed action (*r* = −.35, *p* < .001). Finally, mindfulness was positively correlated with committed action (*r* = .25, *p* < .01).

The initial regression model of the main effects of mindfulness, cognitive fusion and committed action on self‐care behaviour significantly predicted around 7% of the variance in the self‐care maintenance (*F* (3, 161) = 4.39, *p* < .01); however, none of the model variables significantly predicted self‐care maintenance (Table 2). When the interaction terms (i.e. committed action X mindfulness and committed action X cognitive fusion) were added to the model, the model explained about 11% of the variance in self‐care maintenance (*F* (5, 159) = 3.88, *p* < .01). The model variables were all significant except for cognitive fusion (Table 3). The model, however, did not show the nature of the interaction terms in the model.

**TABLE 2:** Linear regression results for the main effects of the study variables on self‐care maintenance models (*N* = 165)

Dependent variable	Predictor	*β*	*SE*	*t*
Self‐care maintenance	Constant	33.62	7.72	4.35**
	Cognitive fusion	−2.34	0.13	−1.88
	Mindfulness	2.34	1.31	1.79
	Committed action	0.19	0.18	1.07

** *p* < .01.

**TABLE 3:** Linear regression results of the main and interaction effects self‐care maintenance (*N* = 165)

Dependent variable	Predictor	*β*	*SE*	*t*
Self‐care maintenance	Constant	−28.75	28.00	−1.02
	Cognitive fusion	0.74	0.49	1.49
	Mindfulness	12.91	5.69	2.26*
	Committed action	2.52	1.01	2.49*
	Committed action X cognitive fusion	−0.04	0.02	−2.08*
	Committed action X mindfulness	−0.39	0.19	−1.98*

* *p* < .05.

Andrew Hayes’ process was then used to examine the nature and direction of the interactions among the study variables in the second regression model. To achieve that, mindfulness and cognitive fusion were divided into low, medium and high levels at 1, 0 and −1 *SD, respectively*. This was done using Hayes’ process. Then, the significance of the effect of cognitive fusion on self‐care maintenance was tested at all possible combinations of various levels of mindfulness and cognitive fusion. Table 4 shows that when the cognitive fusion level was high, the committed action effect on self‐care maintenance was not significant at all levels of mindfulness. However, the committed action effect on self‐care maintenance was only significant at the lower level of mindfulness at the medium level of cognitive fusion and at low and medium levels of mindfulness at the lowest level of cognitive fusion (Table 4).

**TABLE 4:** Conditional effect of committed action on self‐care maintenance at combination of various levels of cognitive fusion and mindfulness (*N* = 165)

Cognitive fusion	Mindfulness	Effect	*SE*	*t*
Low	Low	0.94	0.35	2.63**
Low	Medium	0.58	0.24	2.36*
Low	High	0.22	0.24	0.92
Medium	Low	0.56	0.26	2.10*
Medium	Medium	0.20	0.17	1.15
Medium	High	−0.15	0.23	−0.64
High	Low	0.18	0.28	0.63
High	Medium	−0.17	0.26	−0.67
High	High	−0.53	0.34	−1.54

* *p* < .05.

** *p* < .01.

## 4 DISCUSSION

The purpose of the present study was to examine the potential of using the ACT model to promote self‐care behaviour in persons with heart failure. Specifically, we investigated how committed action affects self‐care maintenance through the interaction with cognitive fusion and mindfulness. We believe this study will help to guide planning future ACT‐based interventions to improve self‐care behaviour in persons with heart failure by understanding the complex relationships among relevant factors. Based on our review, there was no similar study that examined the ACT model and its potential to be used with self‐care behaviour in patients with heart failure.

The current study supported the results from previous literature in many ways. For example, most of our sample showed below optimum self‐care behaviour, with only six patients scoring above the cut‐off point for optimum self‐care maintenance score. This was consistent with the previous literature in that most patients with heart failure have reported below optimal self‐care behaviour (Bidwell et al., 2015; Riegel, Driscoll, et al., 2009), especially in developing countries, such as Jordan (Tawalbeh et al., 2017). These results stress the importance of working to promote self‐care behaviour and the need for evidence‐based interventions that may help in promoting self‐care behaviour in patients with heart failure.

No previous studies have examined the relationship between mindfulness, cognitive fusion and committed action in relation to self‐care behaviour. However, the study variables were associated with self‐care maintenance in the same way they associated with positive behavioural outcomes in previous studies. For example, bivariate analysis showed that mindfulness and committed action were positively correlated with self‐care maintenance as it has been shown to be associated with better social functioning, mental health, vitality, general health and acceptance of pain (Gagnon et al., 2016; McCracken, 2013). Cognitive fusion, on the other hand, was negatively correlated with self‐care maintenance, as it has been shown to be negatively associated with positive health outcomes, such as the quality of life in persons with diabetes (Samadifard & Mikaeili, 2016). Contrary to our expectations, the initial linear regression showed that none of the main effects significantly predicted self‐care maintenance. However, when we added the interaction terms to the model, all main and interaction effects were significant contributors to the model (Table 2). An exception for that was the non‐significant main effect of cognitive fusion.

Looking at the results of the Hayes Process (Table 4) provided a more detailed picture of how committed action interacted with mindfulness and cognitive fusion affecting self‐care maintenance. The effect of committed action on self‐care maintenance at lower levels of cognitive fusion was positive and significant only under lower levels of mindfulness. The most surprising result was that the effect of committed action on self‐care behaviour was significant only at lower levels of mindfulness under lower and medium levels of cognitive fusion. In other words, it was better to have low/moderate mindfulness level under lower levels of cognitive fusion for better effect of committed action on self‐care behaviour. The authors could not locate anything in the literature to explain the behaviour of the committed action variable under different levels of mindfulness. Therefore, there is a need to explain such an unexpected interaction. Although the main effect of the cognitive fusion was not significant, Table 3, cognitive fusion affected the level of self‐care behaviour through its interaction with the committed action.

### 4.1 Implications

The current study has potential implications for both research and practice. Healthcare professionals, such as nurses, involved with providing care to persons with heart failure, need to be knowledgeable about the factors predicting self‐care behaviour. The authors encourage healthcare professionals to keep in mind that self‐care behaviour is a rather complicated phenomenon that is affected by complex relationships and interactions of different variables. The ACT could provide theory‐driven interventions to improve health outcomes in persons with heart failure (Deek et al., 2016). In addition, we believe that the ACT model was very successful in proposing such hidden and complex relationships that affected self‐care maintenance as it was presented in the results of the current study. Thus, regarding the implications to research, the findings of this study open the door for future investigations regarding the applicability of the ACT model‐based interventions in explaining and promoting self‐care behaviours in persons with heart failure. It is highly recommended to examine other potential variables that are derived from the ACT model that may further explain self‐care behaviour in such a population.

### 4.2 Limitations

The current study examined the potential of the ACT model in promoting self‐care behaviour in patients with heart failure. The model suggests various response styles that necessitate a comprehensive plan of action to promote self‐care behaviour in patients with heart failure. However, the current study has two main limitations. First, the study variables represent the ACT model response styles. We believe that more comprehensive and representative variables of the proposed response styles may provide more insight into the interaction among the variables that constitute the ACT model. Second, interventional studies are required to examine how an ACT‐based intervention will affect self‐care behaviour in persons with heart failure. The efficiency of ACT‐based intervention is an empirical question that needs further investigation. In addition, another problem may be the suboptimal Cronbach's alpha of the CFQ and SCHFI V6.2 self‐care maintenance scale. Other limitations of the current study were the use of non‐probability sampling, which might limit the generalizability of the current study outcomes. In addition, the current sample consisted of hospitalized persons with heart failure, including persons with heart failure from outpatient settings, which may make the results more generalizable.

## 5 CONCLUSION

In conclusion, testing the main effect of the proposed variables in this study yielded non‐significant effects. However, when interaction terms were entered into the model, four out of the five terms turned out to be significant. The use of the Andrew Hayes process provided a means to study complex relationships among variables affecting human behaviour that cannot be captured or explained by simple direct effect. Since these interaction terms were suggested by the ACT model, the results of the current study address the potential benefit of using and adopting the ACT model in understanding the underlying psychological processes affecting self‐care behaviours in persons with heart failure. The ACT model stresses working on all response styles in changing human behaviour (Hayes, Luoma, Bond, Masuda, & Lillis, 2006), which provides further support for the potential benefits of adopting the ACT model in promoting self‐care behaviour in patients with heart failure.

## CONFLICT OF INTEREST

The authors declare no known conflicts of interest.
